# Behavioural types correlate with the gut microbiome in juvenile wild and reared gilthead seabream

**DOI:** 10.1098/rsos.250100

**Published:** 2025-10-15

**Authors:** Aina Pons Salom, Eneko Aspillaga, Ignacio A. Catalán, Tomeu Viver, Marco Signaroli, Javier Sanllehi, Amalia Grau, Martina Martorell-Barceló, Margarida Barcelo-Serra, Josep Alós

**Affiliations:** ^1^ Mediterranean Institute for Advanced Studies (IMEDEA, CSIC-UIB), 07190 Esporles, Balearic Islands, Spain; ^2^ LIMIA-IRFAP, IRFAP, CAIB, 07157 Port d’Andratx, Balearic Islands, Spain

**Keywords:** behavioural types, ecological networks, gut microbiome, *Sparus aurata*, juvenile marine fish, gut–brain axis

## Abstract

The gut microbiome influences host behaviour through the gut–brain axis (GBA), a bidirectional network of signalling pathways. Although the GBA has been well studied in humans and other mammals, its role in shaping individual behavioural variation in fish remains largely unexplored. In this study, standardized behavioural tests were conducted on 67 juvenile gilthead seabream (*Sparus aurata*), consisting of 30 wild and 37 reared individuals, across five major behavioural axes—boldness, aggressiveness, sociability, activity and exploration—to determine their behavioural types using linear mixed models. High levels of repeatability of behaviour and consistent behavioural types were observed along the five studied axes. Gut samples from contrasting behavioural types were analysed for diversity, composition and structure using 16S rRNA sequencing. Statistically significant correlations and differences were found between wild and reared groups in both behavioural types and gut microbiome characteristics. These findings provide novel evidence of associations between behavioural types and the gut microbiome in juvenile marine fish, suggesting that gut microbiome may play a role in modulating fish behaviour. While this relationship could involve GBA interactions, further research is needed to confirm such mechanisms. This work could have translational significance for understanding survival, recruitment and life-history evolution in the early life stages of wild fish, as well as improving conservation management of species in both aquaculture and their natural habitats.

## 1. Background

Across nearly the entire animal kingdom, individuals exhibit distinct behaviours compared to other members of their species. When individual behavioural differences are consistent over time and across ecological contexts, these behaviours can be categorized into behavioural types [1,2]. Behavioural types are usually described along five major axes: aggressiveness, activity, sociability, exploration-avoidance and boldness [3]. The presence of different behavioural types along these axes is well documented across taxa, including mammals [4–6], reptiles [7,8], amphibians [9,10], birds [11,12], fish [13,14] and invertebrates [15,16].

In fish, behavioural types have attracted scientific attention over the last few decades due to the importance of individual behavioural differences throughout an organism’s life (across ontogeny) and their implications for different aspects of ecology and evolutionary biology [17,18]. These differences also play a crucial role in survival and recruitment processes within populations [19,20]. However, although evidence supports that genetics, epigenetics, environmental factors and early-life experiences contribute to the development of behavioural types [21–23], the intrinsic mechanisms underlying the presence of distinct behavioural types within the same fish species remain largely unresolved [24]. Over the past decade, the gut microbiome has been proposed as a possible mechanism that could influence fish behaviour [25,26], yet the extent of its contribution to the maintenance of individual differences and ecological consequences remains largely unexplored.

The term ‘gut microbiota’ refers to the community of microorganisms inhabiting the gastrointestinal tract [27], while the ‘gut microbiome’ includes the collective genomes of these microorganisms [28]. In this study, we use the term ‘gut microbiome’ throughout, although we focus specifically on its bacterial component. Within this community, we can differentiate between an allochthonous or transient microbiota, which is free-living and associated with the intestinal content, and an autochthonous or more stable microbiota, attached to the mucosal surface of the intestine [29,30]. The ‘core’, which would be part of the second group, is defined as the portion of the gut microbiome that is found in the majority of hosts within a population or species and may have important biological functions for the host [31,32]. The gut microbiome plays a crucial role in the health, physiology and behaviour of vertebrates [33]. Moreover, this bacterial community can influence the metabolism of neurotransmitters such as serotonin, dopamine and noradrenaline—traditionally associated with behaviour [34,35]—and produce metabolites or microbial compounds that can travel to the brain, modulating brain function and behaviour through the so-called gut–brain axis (GBA) [36]. This complex connection involves a bidirectional communication between the gastrointestinal tract and the central nervous system via the vagus nerve and, in parallel, is linked with the immune and endocrine systems [36,37]. Although the GBA has been extensively studied in humans [37–39], recent years have seen a growing interest in investigating its role in animal behaviour, with most studies focusing on mammals and laboratory models. For instance, antimicrobial-induced dysbiosis has been shown to alter gut microbial composition and increase exploratory behaviour in mice [34]. However, large vertebrate groups such as fish, as well as non-mammalian species and wild animals, remain strongly underrepresented in this research area, despite their ecological relevance and the potential importance of microbiome–gut–brain interactions in these species.

In addition to composition and diversity, the structure of the gut microbiome (i.e. how bacteria co-occur) may also play an important role in shaping behaviour [40,41]. The use of co-occurrence networks in microbiome studies is essential for identifying relationships within the bacterial community and observing influences and interactions with the host [42]. Although direct links between bacterial co-occurrence and animal behaviour remain largely unexplored, network analyses have revealed meaningful ecological patterns in other contexts. For example, co-occurrence analysis has been used to uncover differences in microbial network structure across mosquito species, identifying highly interconnected taxa that may play key roles within the community [43].

In fish, research on the GBA’s role in behaviour is relatively recent and scarce. To our knowledge, the first study mentioning the term ‘gut–brain axis’ in a fish was conducted by Polakof *et al.* [44]. Most studies have been conducted with zebrafish, *Danio rerio* [35,45], as it is one of the animal models used to study the behaviour–gut microbiome connection through the GBA [46]. In this species, the administration of probiotics such as *Lactobacillus rhamnosus* was found to alter certain behaviours and the expression of neurotransmitters at the cerebral level [35]. A more recent study indicates that feeding behaviour affects the intestinal microbiome in various freshwater fish species [41]. Differences in the intestinal microbiome composition between the silver carp, *Hypophthalmichthys molitrix*, and the gizzard shad, *Dorosoma cepedianum*, have been attributed to variations in swimming behaviours, with the former moving between different locations and the latter being more sedentary [25]. Since most experimental studies in this field have been conducted with reared individuals [47,48], our understanding of the GBA and its ecological and evolutionary implications in marine wild fish remains limited, highlighting the need for further research in these species.

To address the existing research gap, here we investigate the potential relationship between the different behavioural types and the gut microbiome in juveniles of the gilthead seabream (*S. aurata*), a marine fish species of interest for fisheries and aquaculture. Conducting standardized laboratory assays, we tested the five major axes of animal personality and related them to the gut microbial community using 16S rRNA gene sequencing. We hypothesized that a link could exist between the behavioural types and the composition, function and structure of the gut microbiome in the juvenile individuals of this case-study species. This work represents an important first step towards exploring potential links between the gut microbiome and behaviour in marine fish. While these findings may be related to GBA interactions, further research is required to confirm the role and relevance of this axis in marine species.

## 2. Methods

To investigate the connection between behaviour and the gut microbiome in wild and reared gilthead seabream, standardized behavioural tests were first conducted in individual aquariums under controlled conditions at the Marine Research and Aquaculture Laboratory (LIMIA-IRFAP), located in Port d’Andratx on the island of Mallorca (Spain). A total of twelve behavioural arenas were set up and used for the experiments, with the specifications detailed in §2.2.

### 2.1. Origins of the studied fish: wild and reared

Two samples of individuals from different origins were used for the study. The wild sample consisted of 30 juvenile gilthead seabream individuals, with an average body size of 12.38 ± 1.12 cm (total length, TL) and a weight of 29.35 ± 6.99 g at the beginning of the experiment. This sample was captured in an enclosed shallow bay in Mallorca Island (Balearic Islands, Spain) using experimental hook-and-line fishing between March and April 2019. Upon capture, the fish were carefully transported in a 50 l oxygenated tank to LIMIA-IRFAP in Port d’Andratx, Mallorca (Balearic Islands, Spain). The wild individuals were directly transferred to the experimental behavioural arenas upon arrival at the laboratory, without intermediate holding, to minimize handling stress and preserve natural behavioural responses. The reared sample was provided by the Institute of Agrifood Research and Technology (IRTA, Spain) in July 2019. Two hundred individuals from the same breeding programme were transported to LIMIA-IRFAP and placed in two 1000 l aquaculture tanks. The fish were kept undisturbed and fed with conventional pellet food (D-2 Optibream AE 1P, Skretting) until the start of the experiments. At the start of the experiments, each fish was individually placed in experimental behavioural arenas for subsequent behavioural testing. From the 200 individuals, 37 were randomly selected (12.8 ± 1.06 cm TL, 30.2 ± 7.89 g) to form the reared sample. Therefore, a total of 67 individuals were used for the study (*n* = 30 wild and *n* = 37 reared). The behavioural scoring period for the wild sample was from 11 March to 23 April 2019, and from 19 July to 22 August 2019 for the reared sample.

### 2.2. Experimental setup for behavioural scoring of the five major axes of behaviour

The design of the behavioural arenas followed the specifications outlined by [49]. Each behavioural arena consisted of a 120 l seawater aquarium with a sand layer bed and a refuge. Behind each tank, a sump station was set up with a water pump, a water heater, and an air stone aerator. The water temperature of the behavioural arenas was electronically controlled to oscillate around 21°C, with a mean and standard deviation of 20.9 ± 0.91°C. The filtration system included two sponges of different thicknesses, a bio-media for biological filtration, and a protein skimmer for the removal of organic compounds. To ensure control of environmental variables, a closed UV-purified seawater re-circulation system was used. The lighting system, consisting of a LED light screen placed at the top of the behavioural arena, was automatically controlled to produce a photoperiod of 12 : 12 h light/dark cycle (see [50] for details).

Individual fish were introduced into the behavioural arenas and kept for 7 days. To maintain the diet each sample was habituated to, the wild sample received three shrimps per day per individual, and the reared sample was fed with 1 g of pellet per day per individual according to the standardized ad libitum feeding protocols established in our laboratory for this species. The initial 3 days were designated as an acclimation period, during which individuals were left entirely undisturbed. For the 4 subsequent days, standardized personality tests for all five behavioural traits were conducted daily to obtain repeated measures for each individual. Each weekly setup comprised four to six fish.

### 2.3. Fish behaviour monitoring and recording

Video recordings of the behavioural arenas were continuously conducted over 4 days of standardized behavioural experiments. The recording setup consisted of a Raspberry Pi computer paired with an 8MP Raspberry Pi Camera Module V2, as detailed in [51]. Behavioural data were extracted using a dual-method approach: manual review and automatic analysis through artificial intelligence. The manual review involved scrutinizing the recordings to tally specific behavioural events. In contrast, the AI analysis utilized a pre-trained Faster R-CNN deep learning algorithm to track individuals within the video frames. This algorithm, validated against over 50 000 frames, achieved a 93% accuracy rate in accurately determining the positions of individuals. Detailed information on the tracking algorithm and its validation can be found in [51].

### 2.4. Standardized behavioural tests

The classical standardized behavioural tests were considered for the five behavioural axes, designed to obtain repeated measures for fish [3]. A detailed description of the experimental procedure is explained in [50]. Following the acclimation period, five tests were conducted, each corresponding to a specific behavioural axis, and were repeated for every individual over 4 consecutive days. The duration of each test was 1 h, except for the activity test, which extended to 2 h.

Activity was described as an individual’s general movement activity in a familiar and safe environment and was measured using the open-field test [52]. The total distance travelled (in pixels) and the area covered (in pixels) by the individual were calculated by the automatic tracking algorithm over 2 h of video recording every morning, before the other tests were conducted [51]. Sociability was defined as the individual reaction to the presence of conspecifics [2]. A conspecific individual similar in size was placed in a small aquarium next to the behavioural arena, and the total amount of time (in seconds) spent inside a 100 pixel (approx. 6 cm) radius from the centroid of the other conspecific was quantified. Exploration was defined as an individual’s reaction to a new situation [2], and was tested using the novel-object test following the detailed methodology described in [50], where a small coloured toy animal figurine was introduced into the arena to assess exploratory behaviour [53]. Aggressiveness, defined as an individual’s agonistic behavioural reaction towards conspecifics [2], was tested using the mirror test [54]. Finally, boldness was defined as the response towards a risky situation [55]. For this purpose, a predator stimulus test was used, where the presence of a predator was simulated by introducing an aquarium gripper as a hostile object into the aquarium for 5 s. After the stimulus, a food item was provided to measure the latency to feed as a measure of boldness in feeding. Regarding the order of the tests, activity was always measured first in the morning, with very little variation in the exact time. Subsequently, the order of the exploration, aggressiveness and sociability tests was randomized to minimize any within-day temporal effects on behavioural measurements. Finally, the boldness test was always conducted at the end of the sequence, as it required feeding the fish prior to the test. Based on this design, any potential effects of time of day on behaviour and/or gut microbiome composition were minimized. For detailed information regarding the standardized tests and metrics used in each of the five classical behavioural axes, see [50].

### 2.5. Behavioural scoring

The aim of the behavioural scoring was to extract a score for each individual and behavioural axis, enabling their classification into different behavioural types. A generalized linear mixed model (GLMM), with the individual as a random effect, was employed to analyse the repeated behavioural data using the MCMCglmm library in R [56]. This statistical approach allowed us to decompose phenotypic behavioural variance into within-individual and among-individual variances and compute the repeatability score (*R*), following the general procedures outlined by [57]. The *R* score is an index used to assess the degree of consistency in among-individual differences over time [58] and is used to confirm the existence of behavioural types. In our models, fish size (mm) and the number of the experimental trials (1 to 5 trials) were included as covariates, and individual ID as a random factor. Following this, adjusted *R* scores for each trait were computed (see details and computation of *R* scores in [50]). Behavioural scores for each individual were obtained from the random effect estimates in the model (i.e. the predicted average behavioural expression for each individual). Each individual was classified according to their behavioural type using the median of its score in bold versus shy, aggressive versus non-aggressive, active versus non-active, social versus non-social and exploratory versus non-exploratory. The parameters of the GLMMs were estimated using a Bayesian approach (Monte Carlo Markov chains, MCMC) using the MCMCglmm R package [56] with default settings for iterations (effective sample size = 13 000), burn-in (3000 first iterations discarded) and thinning (thin = 10) to avoid autocorrelation.

**Figure 1.**
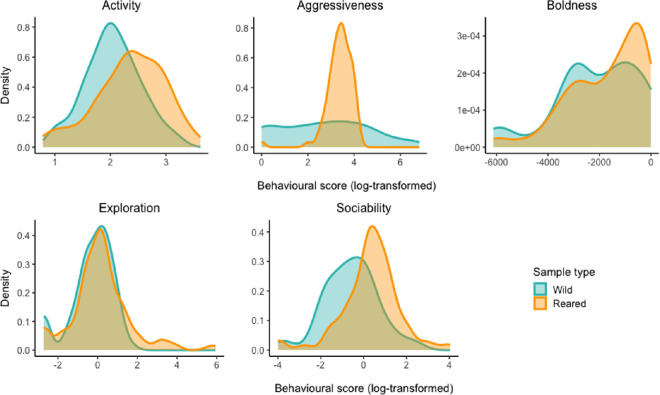
Density plots of individual behavioural scores for the five axes (activity, aggressiveness, boldness, exploration and sociability) in juvenile gilthead seabream, shown separately for reared (orange) and wild (turquoise) individuals. Scores were extracted as individual-level random effect estimates from GLMMs, representing the average behavioural expression of each fish across repeated trials. Aggressiveness scores were log-transformed to normalize their distribution. These scores were used to assign individuals to behavioural types for subsequent microbiome analyses.

The behavioural scores used in this study were originally obtained in [50] and correspond to the same individuals analysed here. These scores were used to classify fish into distinct behavioural types, which were subsequently tested for associations with gut microbiome diversity, composition and structure. The distribution of behavioural scores is shown in figure 1.

### 2.6. Microbial samples, DNA extraction and 16S rRNA analysis

We used the subset of microbiome data corresponding to the wild and reared individuals that were also subjected to the behavioural testing conducted in this study, in order to examine the relationship between behaviour and the gut microbiome. On the last day of behavioural tests, wild and reared gilthead seabream individuals were sacrificed with an overdose of the buffered anaesthetic tricaine methanesulfonate (MS-222; 1 g l^−1^), and their gastrointestinal tracts were collected under sterile conditions and stored in RNA later. The gut microbiome samples from these same individuals were included in the larger dataset analysed in Viver *et al.* [30], which comprised additional samples from fish of different characteristics and years. DNA extraction, 16S rRNA gene amplification, sequencing and sequence processing procedures have been described in detail in Viver *et al.* [30]. Briefly, DNA was extracted using a QIAamp DNA Microbiome Kit (Qiagen, Germany), and sequencing was performed on an Illumina MiSeq platform.

To visualize variation in gut microbiome composition across wild and reared individuals, and between core and non-core fractions, a non-metric multidimensional scaling (NMDS) analysis was performed based on Bray–Curtis dissimilarity matrices calculated from relative abundance data.

### 2.7. Behaviour–microbiome analyses

After determining the behavioural types of each individual and constructing their operational phylogenetic unit (OPU) matrix, correlations between the different behavioural types and the diversity, composition and structure of the gut microbiome in both wild and reared individuals were tested. The associations between the phenotypic behavioural scores and the diversity, composition and structure of the intestinal microbiome were computed with univariate and multivariate analysis. OPUs with zero occurrences were eliminated. All statistical tests were conducted using R v. 4.3.3 [59].

Three different tests were conducted. Diversity and composition tests were performed separately for the core microbiome (OPUs present in at least 80% of the fish) and the non-core microbiome (OPUs present in at least 10% of the fish, excluding the core microbiome). The partitioning between core and non-core fractions, as well as the quantification of the core microbiome by occurrence, followed the approach described in Neu *et al.* [31], where occurrence-based definitions are reported to be the most commonly used in the scientific literature. The 80% threshold was selected to ensure that the core microbiome to identify taxa consistently present across the majority of individuals, while still allowing some margin for natural biological variation, and the 10% threshold for the non-core microbiome minimized the influence of very rare taxa in very few individuals. For the structure tests, the analysis was conducted on all OPUs present in at least 10% of the fish, without separating core and non-core microbiome, as the core alone included too few taxa to construct informative co-occurrence networks. The correlation between OPUs diversity, composition and structure, and the following behavioural types: aggressive–non-aggressive, active–non-active, exploratory–non-exploratory, sociable–non-sociable and bold–shy was analysed.

The correlation structure among behavioural axes in this dataset was previously analysed and published in [50]. Only significant between-individual correlations were found between exploration and sociability, and between exploration and activity. No other significant correlations were detected between behavioural axes. Therefore, each behavioural type was treated as an independent dimension in the microbiome–behaviour analyses conducted in the present study.

#### 2.7.1. Diversity test

Microbial diversity could be defined as a measure of the variety of species present in a community. The microbial diversity was analysed across all the different behavioural types. Three different metrics were computed to assess microbial diversity: (i) the number of OPUs for each individual (number of species or richness), (ii) the Shannon index (diversity), and (iii) the Simpson index. The association between each of these metrics and behavioural scores was analysed using general linear models (GLMs) through an ANOVA. When calculating the GLM with the number of OPUs, a Poisson regression model with a log link function was employed, as this metric represents count data (the number of bacterial species per individual). The model structure was *nsps (number of species)/Shannon/Simpson ~ behav_type + body_weight + week*. Due to data limitations in the behavioural scores of the wild sample, the same *N* was not achieved across all behavioural axes: activity (*n* = 29), boldness (*n* = 15), aggressiveness (*n* = 27), sociability (*n* = 25) and exploration (*n* = 25). An additional general GLM was performed to test the effect of body size and experimental week on the number of species and diversity indices. For this analysis, the activity axis was used, as it had the largest sample size in the wild sample (*n* = 29), allowing for the assessment of the effect using the complete *N*. The experimental week variable was included to assess whether temporal differences could have an effect on gut microbiome diversity. The R packages Vegan [60] and ggplot2 [61] were used.

#### 2.7.2. Composition test

The microbial composition, representing the specific arrangement and abundance of microbial species, was quantified between the different behavioural types using a principal component analysis (PCA) and a redundancy analysis (RDA) controlling for the fish size and experimental week using the R package Vegan [60]. The model structure was *sps (relative abundance matrix of bacterial species) ~ behav_type + body_weight + week*. Additionally, when a statistically significant result was found in the ANOVA of the RDA, a SIMPER analysis was performed to identify the most influential species in the differences observed between behavioural types using Bray–Curtis dissimilarities. The SIMPER analysis quantifies the average contribution of each species to the differences between two groups [62]; in this case, between behavioural types along each behavioural axis. The results of the RDA were graphically represented. Another GLM was employed in this test to assess the effect of body size and experimental week on gut microbiome composition. The activity axis was used since it had the largest sample size in the wild sample (*n* = 29).

#### 2.7.3. Structure test

OPUs present in at least 10% of the individuals were considered in this analysis. A total of 158 OPUs and 194 OPUs were included for the wild and reared samples, respectively.

##### 2.7.3.1. Co-occurrence matrix and probabilistic model

This analysis examined the co-occurrence of bacterial species across different behavioural types. First, a probabilistic model was used to determine the microbial structure associated with each behavioural axis (e.g. aggressive versus non-aggressive), based on the co-occurrence matrix of OPUs identified in each individual [63]. Co-occurrence matrices are commonly used in ecological research to investigate complex species–environment relationships [64] and are valuable for characterizing community structures within microbiomes [65,66]. Accordingly, the presence/absence matrix of OPUs from individuals classified within a given behavioural type (e.g. aggressive type) was used to quantify the co-occurrence patterns, defined as the simultaneous presence or absence of different species within a given behavioural type. The *cooccur* function from the R package *cooccur* [63] was employed to compute both the observed and expected frequencies of co-occurrence between each pair of OPUs and to calculate the probability of obtaining more extreme values of co-occurrence (either higher or lower) by chance. The expected frequency assumes that the distribution of each species is random and occurs independently of other species. The results classify OPU pairs into positive, negative or random (statistically non-significant) associations based on the probabilistic model of species co-occurrence [67].

##### 2.7.3.2. Co-occurrence network construction, visualization, analysis and metrics

Second, based on the co-occurrence data, a co-occurrence network was constructed for each behavioural type. Each node represented an OPU, and associations between nodes were represented as edges. Edges represent connections between nodes with statistically significant co-occurrence between them. The creation and visualization of the co-occurrence networks were conducted with the *igraph* package [68]. A topological analysis of each network was performed to study network structural properties. The network structure was analysed with the label propagation algorithm (LPA) [69], a method used to detect community structure in networks, which is included in the *igraph* package. The communities in the networks consisted of bacteria that tend to co-occur within the gut microbiome of fish exhibiting a specific behavioural type. The number of communities with more than one OPU was counted for each behavioural type to gain insights into the community structure for each behavioural type. Additionally, two metrics, the ‘degree’ and ‘betweenness centrality’, were calculated for each node to analyse the bacterial ecological network properties within each behavioural type. The degree is the total number of associations a node has with other nodes. A higher degree indicates a greater number of direct interactions with other bacteria in the network. Betweenness centrality measures how often a node appears on the shortest paths between other nodes in the network. Higher betweenness centrality values indicate nodes that act as ‘bridges’ or ‘connectors’ and may play a crucial role in linking different parts of the network. To test for statistically significant differences among the network metrics and behavioural types, GLMs were performed using a Poisson distribution, which is suitable for count data. The model structure was: *Degree ~ Behavioural types, family = poisson and Betweeness ~ Behavioural types, family = poisson*.

## 3. Results

### 3.1. General results of the gut microbiome composition

The gut microbiome data used in this study correspond to the same subset of wild and reared gilthead seabream individuals included in Viver *et al.* [30], for which behavioural data were also collected. The diversity of the gut microbiome of the 67 juvenile gilthead seabreams (*n* = 30 wild and *n* = 37 reared) was analysed using 16S rRNA gene amplicon sequencing. A total of 6 618 979 high-quality 16S rRNA gene sequences were clustered into 1656 OPUs. After removing the OPUs with no occurrences in any fish included in this study, 695 OPUs were obtained for the wild sample and 935 OPUs for the reared sample. The taxonomic composition differed between the wild and reared samples, with Proteobacteria (*n* = 762 bacterial counts assigned to this phylum) being the most abundant phylum in the wild sample (68%), followed by Firmicutes (*n* = 470) with 15%, Actinobacteriota (*n* = 202) with 9% and Bacteroidota (*n* = 119) with 3%. Notably, the most abundant OPU was *Ralstoni*a sp. 1 (OPU 0522) with a relative average abundance of 28.3 ± 27.8% (s.d.), followed by *Roseovarius* sp. 2 (OPU 0867), with an average abundance of 4.1 ± 4.9% and *Methylorubrum* sp. 1 (OPU 0962) with an average abundance of 2.5 ± 1.9% (electronic supplementary material A, table A1). In the case of the reared sample, Firmicutes (*n* = 470) was the most abundant phylum, representing 77% of the total abundance, followed by Proteobacteria, with 17%. The other phyla were present in smaller proportions. The three most abundant OPUs represented more than 70% of the total composition, with *Lactobacillus* being the most abundant. At the OPU level, the species *Lactobacillus* sp. 25 (OPU 0036) had an average abundance of 37.8 ± 16.2%, followed by *Lactobacillus* sp. 1 (OPU 0011) and *Lactobacillus* sp. 6 (OPU 0016), with average abundances of 23.8 ± 10.1% and 10.4 ± 4.5%, respectively (electronic supplementary material A, table A4).

The core microbiome, defined by the OPUs present in more than 80% of the individuals, comprised 8 OPUs in the wild sample and 7 in the reared sample. Notably, both cores shared only a single species, OPU 0053, *Streptococcus* sp. 1, a species phylogenetically close to *Streptococcus castoreus*. Three phyla formed the core microbiome in the wild sample, with Proteobacteria (62.5%), followed by Firmicutes (25%) and Actinobacteriota (12%). The most abundant OPU in the core microbiome of the wild individuals was *Ralstonia* sp. 1 (OPU 0522), with an average abundance of 59.6 ± 23.9%, while the rest of the OPUs had average abundances below 10% (electronic supplementary material A, table A2). In the reared sample, the core was entirely composed of the phylum Firmicutes, with five members from the genus *Lactobacillus* sp., one from the genus *Streptococcus* sp. and one from the genus *Weissella* sp. One OPU had a higher abundance within the core, in this case, *Lactobacillus* sp. 25 (OPU 0036), with an average abundance of 49.8 ± 7%. The second most abundant OPU, with an average abundance of 32.4 ± 4.4%, was *Lactobacillus* sp. 1, OPU 0011 (electronic supplementary material A, table A5).

The non-core microbiome, defined by the OPUs present in more than 10% of the individuals, excluding the core, comprised 150 OPUs in the wild sample and 187 OPUs in the reared sample. Its composition differed markedly between the two groups. In wild individuals, the three most abundant species belonged to the phylum Proteobacteria: *Roseovarius* sp. 2 (OPU 0867; 8.1 ± 8.8%), *Photobacterium* sp. 1 (OPU 0617; 4.5 ± 9.7%) and an uncultured bacterium from the family Legionellaceae (OPU 0768; 4.3 ± 10.4%), with an average abundance of 4.3 ± 10.4% (electronic supplementary material A, table A3). In contrast, the reared sample was dominated by *Labrenzia* sp. 1 (OPU 0950; 17.61 ± 23.68%), followed by *Lactobacillus* sp. 12 (OPU0022; 5.0 ± 4.6%) and *Lactobacillus* sp. 14 (OPU 0024; 4.3 ± 4.2%) (electronic supplementary material A, table A6).

**Figure 2.**
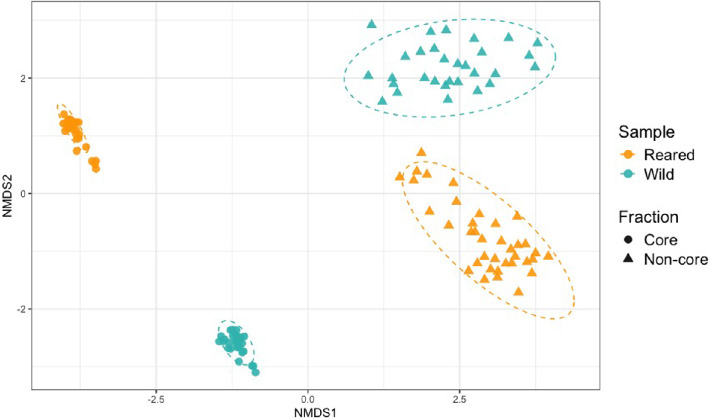
NMDS ordination plot based on Bray–Curtis dissimilarity showing gut microbiome composition across wild and reared samples, and between core and non-core fractions, in juvenile gilthead seabream. Each point corresponds to the gut microbiome composition of one individual fish for a given fraction. Colours indicate sample origin (reared in orange, wild in turquoise), while shapes distinguish the microbiome fraction (core = circles, non-core = triangles). Ellipses represent 95% confidence intervals around group centroids.

The NMDS revealed that gut microbiome composition differed clearly between wild and reared individuals, and also between the core and non-core fractions (figure 2). This indicates distinct microbial community profiles associated with both origin and microbiome fraction.

### 3.2. Univariate analysis: number of species, diversity indices and behavioural axes

Through a univariate analysis (GLM), the relationship between microbial diversity and the five behavioural axes in gilthead seabream was investigated. In the case of the core microbiome, no statistically significant differences were found between the number of species, Shannon index and Simpson index across the different behavioural types, body size and experimental week, neither in the wild sample nor in the reared sample (*p* > 0.05; electronic supplementary material B, tables B2 and B3).

The results were different for the non-core microbiome, where statistically significant associations were found between the number of species and the behavioural axes (electronic supplementary material C) of activity (estimate = 5.05 ± 0.54 s.e. for active beh. type, 0.12 ± 0.06 for non-active beh. type, *p* = 0.041) and exploration (5.02 ± 0.53 for exploratory beh. type, 0.18 ± 0.06 for non-exploratory beh. type, *p* = 0.003; electronic supplementary material B, table B2) in wild individuals. In more detail, more active and exploratory fish showed a higher average number of species in the wild sample. For the reared sample, statistically significant differences were found between the boldness axis and the number of species (4.80 ± 0.34 for bold beh. type, −0.10 ± 0.04 for shy beh. type, *p* = 0.037; electronic supplementary material B, table B3), with the shyest individuals having a lower number of bacterial species. For the non-core microbiome, body size and experimental week appeared to have no impact on the species number, Shannon index or Simpson index (*p* > 0.05 electronic supplementary material B, table B3).

### 3.3. Multivariate analysis: gut microbiome composition and behavioural axes

**Figure 3.**
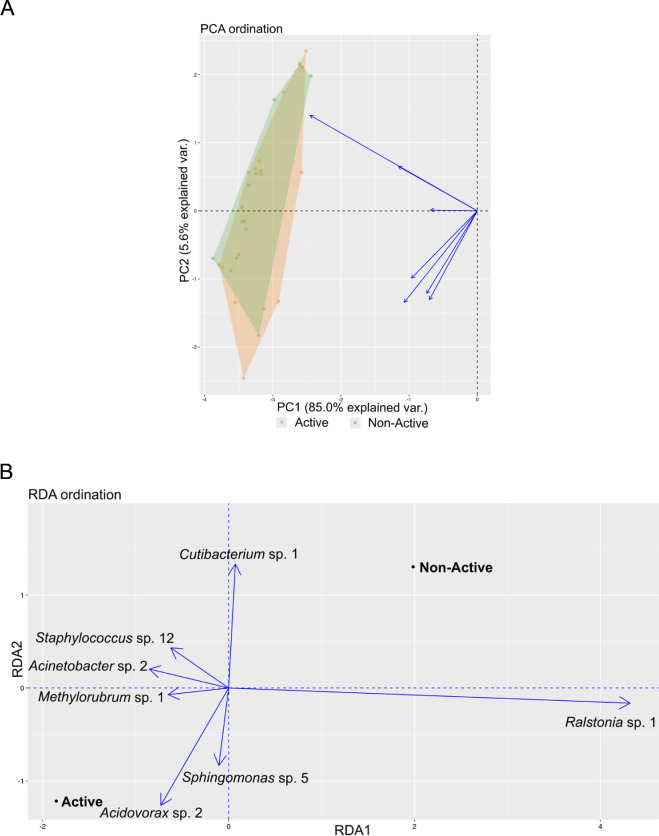
(A) PCA ordination showing the distribution of the core gut microbiome composition of wild individuals, grouped by behavioural type (active individuals in orange, and non-active individuals in green). Each point represents an individual, and each arrow represents the contribution of a specific OPU to the variance. (B) RDA ordination plot displaying the relationship between the core gut microbiome composition and the activity-related behavioural types (active and non-active) of wild individuals. The arrows indicate the contribution of each influential species in relation to the behavioural type.

**Figure 4.**
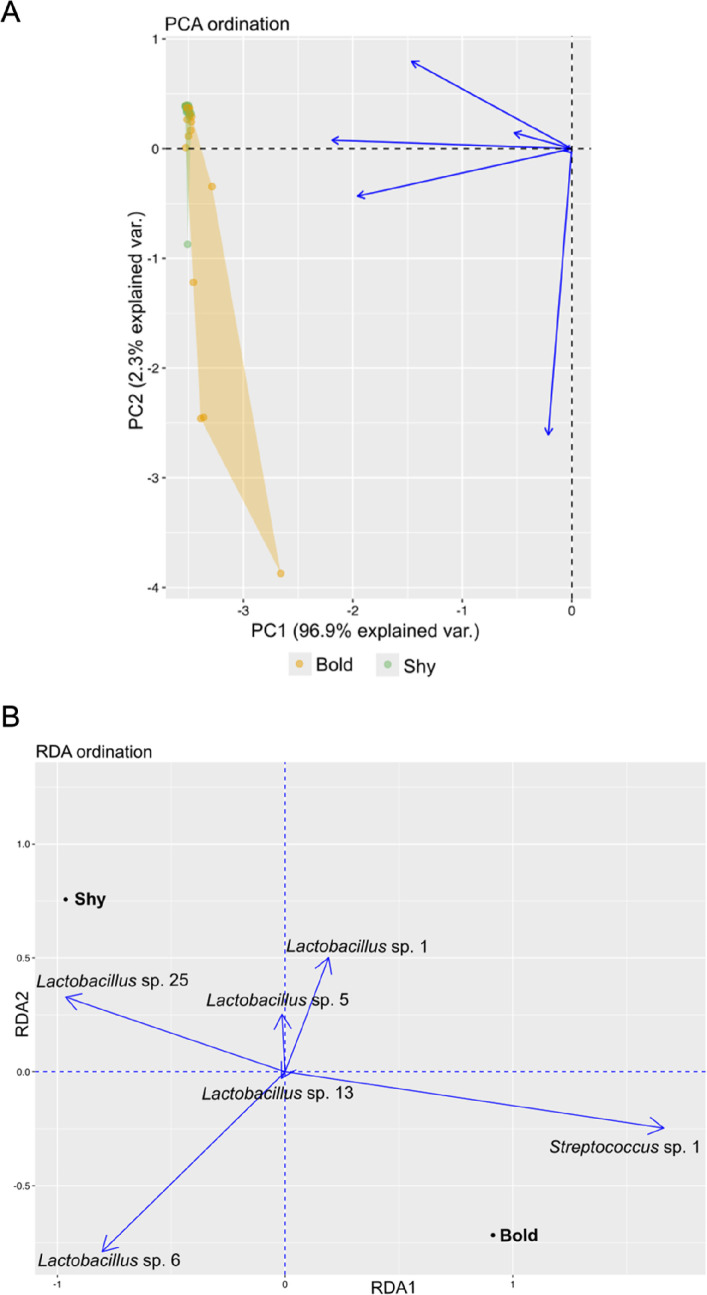
(A) PCA ordination showing the distribution of the core gut microbiome composition of reared individuals, grouped by behavioural type (bold individuals in orange, and shy individuals in green). Each point represents an individual, and each arrow represents the contribution of a specific OPU to the variance. (B) RDA ordination plot displaying the relationship between the core gut microbiome composition and the boldness-related behavioural types (bold and shy) of reared individuals. The arrows indicate the contribution of each influential species in relation to the behavioural type.

To study the relationship at the community level, the correlation between gut microbiome composition, based on the relative abundances of OPUs, and behaviour was examined. For better understanding, PCAs and RDAs were performed across all behavioural types. The results differed from the univariate analysis. Statistically significant associations between the activity axis and gut microbiome composition were found in the core of wild individuals (Var. = 86.24, *F* = 3.06, *p* = 0.045; electronic supplementary material B, table B4). Specifically, certain OPUs belonging to the core were more abundant in active individuals: *Acidovorax* sp., *Methylorubrum* sp., and *Sphingomonas* sp., while others like *Cutibacterium* sp. were more abundant in non-active individuals (figure 3). On the other hand, statistically significant correlations were found between the boldness axis and gut microbiome composition in the core of the reared sample (Var. = 97954341, *F* = 5.95, *p* = 0.028; electronic supplementary material B, table B5), with species like *Streptococcus* sp. being more abundant in bold individuals (figure 4), and a species of the genus *Lactobacillus*, phylogenetically close to *Lactobacillus aviarius*, being more abundant in shy individuals (figure 4). For the non-core microbiome, the results were different: behaviour did not seem to correlate with microbiome composition in the wild sample (*p* > 0.05; electronic supplementary material B, table B4) nor in the reared sample (*p* > 0.05; electronic supplementary material B, table B5).

A statistically significant association was found between body size and the non-core microbiome composition in wild individuals (Var. = 71.08, *F* = 2.26, *p* = 0.002; electronic supplementary material B, table B4) but not in reared individuals (*p* > 0.05; electronic supplementary material B, table B5). Body size and experimental week appeared to have no influence on core composition in wild and reared individuals (*p* > 0.05; electronic supplementary material B, tables B4 and B5). Moreover, the composition of the non-core microbiome was significantly affected by the experimental week in wild individuals (*p* < 0.05; electronic supplementary material B, table B4), considering all behaviour axes except for the activity axis, where no correlation was found (*p* = 0.203; electronic supplementary material B, table B4).

### 3.4. Gut microbiome structure: behavioural axes, co-occurrence analysis and co-occurrence networks

**Figure 5.**
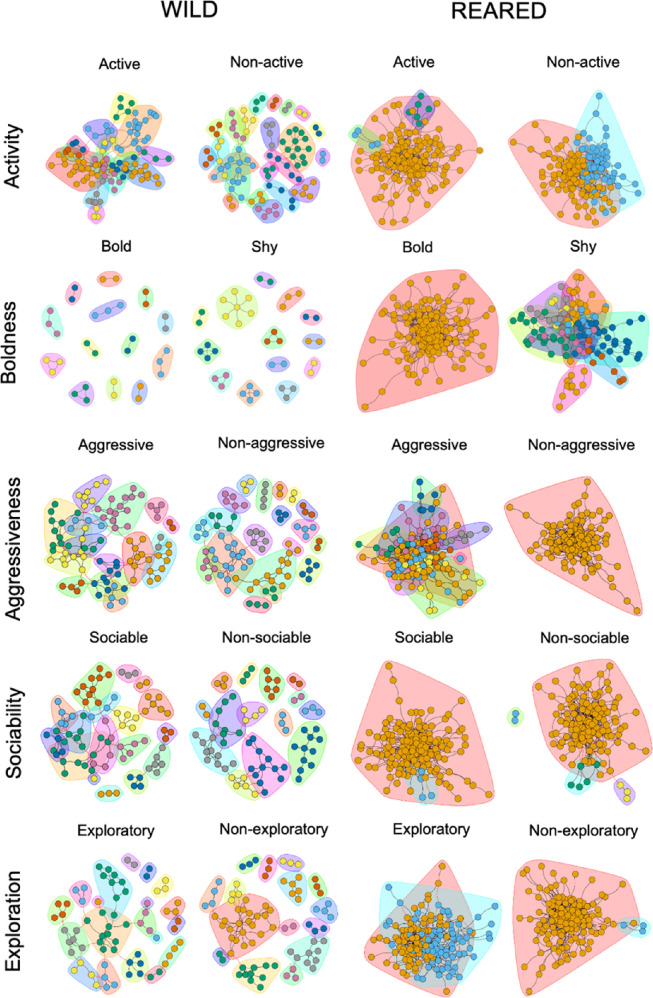
Co-occurrence network graphs of the gut microbiome in all behavioural types in the wild and reared samples of gilthead seabream. Each behavioural type is represented by a graph. From left to right, the first behavioural axis (activity) includes active and non-active fish from the wild sample, followed by active and non-active fish from the reared sample. The same order applies to the other behavioural axes. Each node represents an OPU, and the colour of each node represents its membership within a community, as detected by the LPA. The edges show co-occurrence between OPUs within each behavioural type. Black edges indicate statistically significant co-occurrence between nodes of the same community, while red edges indicate statistically significant co-occurrence between nodes from different communities. The colours of the communities are assigned independently in each network and do not correspond to the same community across different graphs. Within each network, they are used to visually differentiate communities detected by the LPA.

The analysis of the gut microbiome structure revealed differences between the different behavioural types (electronic supplementary material B, tables B6 and B7). In the wild sample, the number of communities detected among behavioural types was similar across the boldness, sociability and exploration axes, with an average of 13.7 ± 0.8 communities (electronic supplementary material B, table B6). However, this was not the case for the activity and aggressiveness axes, where differences in the number of co-occurrence communities were found (electronic supplementary material B, table B6). In active fish, fewer communities were found within the co-occurrence network compared to non-active fish (electronic supplementary material B, table B6). Additionally, active fish exhibited, on average, a higher degree and betweenness centrality than non-active fish (electronic supplementary material B, table B6). The network of active individuals was less structured and had more interconnections or associations between nodes (OPUs) (figure 5). The nodes (each OPU) in the network of an active individual had, on average, twice the associations with other nodes or OPUs in the network (electronic supplementary material B, table B6). Similarly, fewer communities were found in aggressive fish, and on average, a higher degree and higher betweenness centrality were observed compared to non-aggressive fish (electronic supplementary material B, table B6). In other words, aggressive fish exhibited a less structured but more connected co-occurrence network (figure 5). A relationship was found between degree and behaviour across all axes (*p* < 0.05; electronic supplementary material B, table B6) except for sociability in wild individuals (*p* > 0.05; electronic supplementary material B, table B6). Similarly, a significant relationship was found between betweenness centrality and behaviour across all axes in wild individuals (*p* < 0.05; electronic supplementary material B, table B6).

In the reared sample, a lower number of communities across the behavioural axes was found (electronic supplementary material B, table B7). All axes shared a roughly equal number of communities, between 1 and 3 communities with more than two members, except for the boldness axis, which visually showed a clear and distinct pattern between bold and shy individuals, as it differed the most in the number of communities (electronic supplementary material B, table B7; figure 5). Shy individuals presented 11 communities, while bold individuals had only 1 (electronic supplementary material B, table B7). In this case, shy individuals exhibited a more fragmented and varied community structure and bold individuals exhibited a more densely connected network (figure 5). Regarding degree, bold fish had, on average, a much higher degree (electronic supplementary material B, table B7). However, the opposite was true for betweenness centrality, where shy fish had a higher average (electronic supplementary material B, table B7). In other words, the gut microbiome co-occurrence network of shy individuals in the reared sample had more nodes that connected different parts of the network or more nodes acting as ‘bridges’ than bold individuals (electronic supplementary material B, table B7). For the other axes, a relationship was found between degree and behaviour (*p* < 0.05 in all axes; electronic supplementary material B, table B7), as well as between betweenness centrality and behaviour (*p* < 0.05 in all axes; electronic supplementary material B, table B7).

## 4. Discussion

In this work, we investigated the relationship between behavioural types and the microbial community present in the gastrointestinal tract of wild and reared gilthead seabream by combining standardized behavioural tests and 16S rRNA gene sequencing of the gut microbiome. The results of this study revealed multiple associations between the diversity, composition and structure of the intestinal microbiome and behavioural types in the gilthead seabream. Our findings suggest a potential connection between microbiome and behavioural types in marine fish, highlighting the poorly understood nature of this relationship. To our knowledge, this is the first study examining this relationship in the gilthead seabream. The study of the relationship between behaviour and gut microbiome is relatively new, although it is rapidly growing, and it is being investigated in different fish species.

The intestinal microbiome found in our species case study was largely composed of Proteobacteria, Firmicutes, Actinobacteria and Bacteroidetes. This composition resembles a typical gut microbiome composition for gilthead seabream, as described in various studies [70,71]. Differences were observed in the composition of the gut microbiome between the wild and reared samples. Rearing conditions involve the manipulation of various factors, such as the environment, social interactions and diet, which influence the gut microbiome [70]. In this study, we found that the total number of OPUs differed between the two samples, with 690 OPUs identified in the wild sample compared to 932 in the reared sample, as reported in [30]. Our results contrast with the lower numbers of operational taxonomic units documented in aquaculture fish [71], which are generally expected due to the characteristics of the feed and controlled environmental conditions [72,73]. Although a common trend in the scientific literature suggests a reduction in gut microbiome diversity in fish under captive conditions [74], recent studies show significant variability in the results [75]. This variability is likely due to the high diversity of species studied, differences in methodologies and other influencing factors such as diet [70,71].

In wild individuals, *Ralstonia* sp. 1 (OPU 0522), an OPU phylogenetically close to *R. mannitolilytica*, was the most abundant species within the gut microbiome. Some members of the genus are reported in the scientific literature as among the most abundant in the gut microbiome of various fish species [73,76,77]. Interestingly, in other studies of gilthead seabream, this OPU has been identified as having potential beneficial functions for the host, such as antibacterial activity and the production of various compounds and metabolites [78]. However, it has also been described as pathogenic in juvenile olive flounder, *Paralichtys olivaceus* [79]. It was among the most abundant genus in the intestinal microbiome in birds, according to the study by [80]. Another abundant species found was *Roseovarius* sp. 2 (OPU 0867), closely related to *R. aestuariivivens*, a species isolated from marine environments and marine animals [81]. A species of the same genus has been documented to increase survival and immunity in the swimming crab [82]. Other species of the genus can also cause diseases in fish [83]. Another abundant OPU found was *Methylorubrum* sp. 1 (OPU 0962), an OPU close to *M. thiocyanatum*. The genus is known for its beneficial functions in plants [84] and has also been reported in fish as part of the gut microbiome [85]. Despite the diverse functions reported for these bacteria in other studies, the high abundance of these species observed in our research may suggest a potential important role for the host and the health of wild gilthead seabream.

The reared sample showed a microbial community distinct from that of wild individuals, with a notable dominance of members of the genus *Lactobacillus*, especially *Lactobacillus* sp. 25 (OPU 0036), followed by *Lactobacillus* sp. 1 (OPU 0011) and *Lactobacillus* sp. 6 (OPU 0016), as found in [30]. The first species is phylogenetically close to *Lactobacillus aviarius*, which has been identified as part of the allochthonous microbiota in *Salmo salar* fed with a commercial diet [86]. In birds, it has been described as part of the gut microbiome and recognized as a biomarker of good host health [87]. The gut microbiome of reared individuals was dominated by *Lactobacillus* species, which together accounted for more than 70% of the total community. Different *Lactobacillus* species were among the most abundant in both the core and non-core fractions, raising a debate related to the hypothesis explained in [30], which suggests that bacteria from the commercial dry feed would become part of the allochthonous community of the gut microbiome in this species. Consequently, much of the bacterial composition detected by sequencing would originate from the ingested feed, thereby questioning the findings of previous studies and the sampling procedures. Beyond the potential influence of the administered diet, the high abundance of these bacteria may also suggest important implications for the overall health of aquaculture fish. This aligns with the widely studied probiotic potential capacity of various species within the *Lactobacillus* genus, which have demonstrated multiple benefits, including their ability to alleviate stress, anxiety and altered behaviours [88].

The thresholds selected for defining core (>80% occurrence) and non-core (>10% and <80% occurrence) microbiome fractions were chosen to balance the inclusion of taxa consistently present across individuals while reducing the influence of rare taxa. This occurrence-based approach is commonly applied in microbiome studies [31], but it is important to acknowledge that there is currently no universal standard for defining core microbiome. The choice of thresholds can influence the detection of patterns and should therefore be interpreted with caution. In our study, the core fraction provided insights into taxa that are stably associated with the host across individuals, whereas the non-core fraction captured more dynamic components of the microbiome, which may be more responsive to environmental or behavioural variation. Future work should further explore how different threshold choices impact the observed associations between behaviour and gut microbiome. Importantly, there is also a need to establish and follow a more standardized approach for defining and partitioning the core and non-core microbiome across studies.

Only one bacterial species was shared between the core of the wild sample and the reared sample, OPU 0053, *Streptococcus* sp. 1, suggesting that it may serve an important function within the core gut microbiome of gilthead seabream. The species found is phylogenetically close to *S. castoreus*, an uncommon species documented in rodents, part of the resident gut microbiota, but occasionally acting as an opportunistic pathogen [89]. Other species of this genus, such as *S. agalactiae*, are considered pathogenic and have been found responsible for alterations in the microbiome–gut–brain axis and a dysregulation of swimming behaviour in Nile tilapia, *Oreochromis niloticus* [90]. Although several species of the genus are known to be harmful to animals and humans, some belong to the resident fish gut microbiome [72] and others may have a probiotic function [91,92].

In relation to the aim of the study, associations were found between specific behavioural axes and the number of OPUs or bacterial species. In the wild sample, active and exploratory individuals exhibited a greater number of bacterial species than non-active and non-exploratory individuals in the non-core gut microbiome bacterial community, suggesting that these behaviours might be associated with a richer and more diverse microbiome. One possible explanation could be that they have more interactions with others and greater exposure to different environments in their natural habitat. In relation to this, it was found that microbial diversity was higher in a population of nine-spined stickleback, *Pungitius pungitius*, that use a broader niche [93]. Consistent with this, Florkowski & Yorzinski [94] suggested that high-exploratory birds have a more diverse gut microbiome due to occupying wider spatial ranges, which allows them to acquire different types of bacteria from various environments. Additionally, it is known that more active humans, who exercise more, have a more diverse gut microbiome [95]. However, the Shannon and Simpson diversity indices did not show statistically significant differences across the behavioural axes, which may indicate that, while the number of species can vary due to the nature of certain behaviours, there is an even distribution of these species within the microbiome, which is relatively consistent across different behavioural axes. Other studies reported a shift in the statistical significance of the relationship between Shannon and Simpson diversity indices of the gut microbiome and behaviour, depending on different time points. In some instances, the relationship was statistically significant, whereas in others, it was not [96]. This could be related to our results, as we only have data from one time point, and it has been observed in the literature that changes in the gut microbiome can occur rapidly across time in animals [97] and humans [98]. Even in controlled environments, such as the reared sample in this study, statistically significant differences in the number of species were still found in relation to the boldness axis in the non-core gut microbiome. This could suggest that bold individuals had access to more diverse food items compared to individuals who do not take risks [99]. As a result, bold individuals could be exposed to more bacterial sources. Conversely, a study did not find a relationship between bold–shy individuals and gut microbiome diversity in Mongolian gerbils [100], and the same was observed in *D. rerio* [46]. The discrepancy in these differing results could be due to various factors, such as differences between the species studied, environmental conditions, diverse methodological approaches in behavioural tests, variations in statistical analyses or differences in how the gut microbiome is analysed. For instance, we differentiate between the core and non-core microbiome, whereas other studies do not. All these factors may influence the robustness of the results. Therefore, in studies examining the relationship between behaviour and the microbiome, it is crucial to ensure that all variables are clearly defined, standardized, and follow a well-established protocol.

Interestingly, no relationship was found between core diversity and behaviour within each sample (wild and reared), suggesting that, under the tested conditions, the core microbiome is relatively consistent across individuals of similar environmental background and developmental stage. However, the marked differences in core composition between wild and reared individuals observed in this study do not support the existence of a stable core gut microbiome across gilthead seabream as a species. Most previous studies investigating the core microbiome in gilthead seabream have focused on aquaculture settings [101,102], whereas the core microbiome of wild individuals remains largely unexplored to date. This is particularly important, as the core microbiome in wild species is still poorly studied, and most existing studies report the presence of a stable core gut microbiome only in certain model or farmed species, such as *D. rerio* [46] and farmed *S. salar* [103]. Furthermore, our findings highlight the need to investigate how environmental factors, behaviour and the GBA interact and influence each other in wild marine species.

The relationship between behaviour and the gut microbiome is often reflected in the composition of the microbial community or the relative abundance of certain bacterial species. A study in honeybees showed that differences in gut microbial composition were associated with variations in both health and behaviour [104]. In the Seychelles warbler, *Acrocephalus sechellensis*, gut microbiome composition, rather than diversity, was associated with individual survival in the wild [105]. In mammals, the gut microbiome has also been linked to behavioural traits. For example, bold rodents showed higher relative abundances of *Odoribacter* sp. and *Blautia* sp., while *Streptococcus* sp. was more abundant in less social wild mammals [100,106]. Although this connection has been investigated in multiple animal taxa, there is limited research available in fish. For instance, in *D. rerio*, changes in the abundance of specific bacterial species depending on host behaviour have been documented [107]. In this study, we found correlations between gut microbiome composition and behavioural types in both wild and reared gilthead seabream. Specifically, a different relative abundance was found in the species that form the core in active and non-active individuals in wild individuals of gilthead seabream. Species such as *Acidovorax* sp., *Methylorubrum* sp., and *Sphingomonas* sp. were more abundant in active individuals, while species like *Cutibacterium* sp. were more abundant in non-active individuals. In the boldness axis of reared fish, shy individuals had a higher abundance of a *Lactobacillus* sp., while bold individuals showed increased abundance of a *Streptococcus* sp. Overall, these findings suggest that behaviour–gut microbiome interactions may have important ecological and health-related implications in both wild and reared gilthead seabream.

Recent research emphasizes that the GBA could be influenced not only by the diversity and abundance of microbial species but also by their co-occurrence and interactions within the microbiome [40,108,109]. Here, we found that some communities of OPUs that co-occur together could be related to distinct behavioural types. The number of communities present in the co-occurrence networks differed between active and non-active and aggressive and non-aggressive wild individuals, suggesting that the structure of the gut microbiome—how bacteria interact and coexist—is related to behavioural type. In reared individuals, the number of communities was lower across all behavioural axes, except for boldness, where we found that the gut microbiome of shy individuals had more communities and interacted differently from bold individuals. It is reasonable to assume that the structure of the gut microbiome could differ from that of wild individuals of the same species due to the conditions of captivity and the diet of reared individuals. Importantly, this reduced network complexity in reared fish cannot be simply attributed to lower microbial richness, as these individuals exhibited a higher number of OPUs than their wild counterparts. This suggests that the observed differences in network structure may instead reflect disrupted microbial interactions or altered community assembly processes associated with rearing conditions. Furthermore, we found differences in degree and betweenness centrality according to behavioural type. In active and aggressive wild individuals, we found higher degrees and betweenness centrality, indicating that these individuals had a less structured but more connected network than their counterparts in other behavioural types. In the reared sample, bold individuals had a higher degree but lower betweenness centrality, resulting in a less structured yet more connected network. These multiple patterns found could suggest that a specific behavioural type might be associated with a particular gut microbiome structure in gilthead seabream. However, further studies are needed to better understand the underlying mechanisms.

In this study, we also tested the effect of body size and the experimental week on the diversity, composition and structure of the gut microbiome, as these are variables that are worth considering when analysing the intestinal bacterial community [96,110]. Neither the body size of the individual nor the experimental week had a statistically significant effect on the diversity of the gut microbiome in gilthead seabream. Similarly, no influence of body size on gut microbiome diversity was found in different fish species in the Yellow River [111]. However, other studies have found a relationship between gut microbiome diversity and individual size in lake anchovy, *Coilia ectenes taihuensis* [112]. The similar body size of the tested individuals and the fact that all were juveniles could explain the absence of this relationship in the present study. Replicating the experiment with adult individuals of a larger body size would give us a broader and more accurate understanding of the potential relationship. However, despite the lack of variability in the size of the individuals, body size did influence the composition of the microbial community that forms the non-core in wild individuals but not in reared ones. This result, and the lack of influence of body size on the core microbiome within each sample (wild and reared), suggests that the core fraction is relatively consistent across individuals of similar size and developmental stage under the same environmental conditions. However, given that the core microbiome differed substantially between wild and reared fish in our study, sharing only one OPU, these results do not support the existence of a stable core microbiome across gilthead seabream in general. Rather, they highlight the influence of environmental and rearing conditions on shaping the core gut microbiome. In contrast, the non-core bacterial community appeared more variable and responsive to both body size and experimental week in wild individuals. The lack of relationship between body size and gut microbiome composition in reared individuals could be due to the controlled environment, social interactions and artificial diet [70]. In the scientific literature, it has been found that differences in body size could result in a different composition of species within the gut microbiome [112]. Another factor to consider is that the experimental week was also related to the composition of the non-core in wild individuals but not in reared ones. Similarly, the artificial environment and captivity conditions could explain these findings. Therefore, considering experimental time and body size would be especially relevant for studying the relationship between behaviour and microbiome in wild animals.

One of the important aspects to consider when studying the gut microbiome is implementing a fasting period, as discussed in [30], depending on the objective of the study. This is particularly relevant in aquaculture conditions, where it has been suggested that without a fasting period, much of the bacterial community detected would come from the diet and not from the resident or autochthonous community, which would be captured only after a short fasting period of at least 2 days. In our case, no fasting was imposed. However, given the meticulous protocol followed, the intestinal content and bacteria adhered to the mucosa were considered, so we would be capturing part of the mucosal autochthonous community as well as the allochthonous portion that could belong to the diet. Nevertheless, this is an aspect that should be considered in future studies to obtain more precise results. Related to this, it is important to consider the effect of diet on the gut microbiome, not only under aquaculture conditions. Diet is a key factor shaping the gut microbiome in wild animals as well [70,73,97,113]. However, in wild fish, the high variability and limited control over exact dietary intake complicate direct testing of this hypothesis. In our study, it is therefore very likely that the diet of wild individuals influenced the composition of the gut microbiome. This factor should be carefully considered in future studies exploring microbiome–behaviour relationships in wild fish.

Another point to consider for future research would be taking measurements from the same individuals at different times. By doing this, the relationship between the microbiome and behaviour could be better investigated, and the mechanisms at play could be elucidated. However, this is difficult and labour-intensive, especially when studying wild individuals, as fish with relatively small intestines do not allow for non-invasive sampling to sequence the 16S rRNA gene. Age and body size are also relevant factors. Age has also been identified as a factor in different studies, with differences found between the gut microbiome of juveniles and adults [113]. Therefore, including adult individuals would be interesting for disentangling the connection investigated in this study. The sampling effort for bacterial species coverage is another important aspect, as too small a sample size could distort the results. For gilthead seabream, it is estimated that nine biological samples per group cover most bacterial species that could be detected [114]. Despite the limitations encountered, the results are robust and provide valuable insights for future research.

## 5. Conclusion

This study provides initial evidence of a potential link between gut microbiome composition and behaviour in wild marine fish, which may be influenced by GBA interactions. However, further research is needed to clarify the mechanisms driving this relationship. Our results, while qualitative and to be interpreted cautiously, suggest that both individual bacterial taxa and their interactions within the gut microbiome may contribute to host processes related to behaviour. These findings underscore the complexity of host–microbiome interactions and highlight the importance of carefully designed methodologies to unravel these connections. Moreover, manipulating the microbiome emerges as a promising tool to explore host–microbiome interactions and improve aquaculture management. This study establishes a baseline for future research in both wild and reared marine species, allowing us to extend our results to an ecological context and improve the biology and management of species in both their natural habitats and in aquaculture.

## Data Availability

All relevant data and resources can be found within the article and its supplementary material [115].
